# Chronic alcohol exposure parametric effects on anxiety- and pain-related behaviors in adult rats

**DOI:** 10.1016/j.alcohol.2026.01.157

**Published:** 2026-01-29

**Authors:** Maria E. Secci, Loren Johnson, Thomas Lobell, Sydney Long, Lillian Shepherd, Nicholas W. Gilpin, Elizabeth M. Avegno

**Affiliations:** a Department of Physiology, Louisiana State University Health Sciences Center School of Medicine, New Orleans, LA, 70112, USA; b Alcohol and Drug Abuse Center of Excellence, Louisiana State University Health Sciences Center School of Medicine, New Orleans, LA, 70112, USA; c Veterans Affairs Southeast Louisiana Healthcare System, New Orleans, LA, 70112, USA

**Keywords:** Alcohol, Dependence, Withdrawal, Vapor exposure, Nociception, Anxiety, Rats

## Abstract

Many animal models of alcohol dependence utilize forced alcohol exposure, including chronic intermittent exposure to alcohol vapor, to induce high blood alcohol concentrations (BACs) and withdrawal-associated behaviors similar to those seen in clinical contexts. Chronic alcohol exposure and withdrawal are especially important in influencing the expression of negative symptoms (e.g., negative affect and pain), which, in turn, increase alcohol consumption. However, cessation of chronic alcohol vapor exposure does not always lead to those “canonical” withdrawal behaviors in rodents. Environmental and genetic factors may modulate alcohol effects on behavior during withdrawal. Here, we used retrospective data analysis to determine associations between alcohol vapor exposure parameters (e.g., BACs, duration of exposure) and anxiety- or pain-like behavior in adult male and female Wistar rats. Our results indicate that specific vapor exposure parameters are predictive of thermal hyperalgesia in Wistar rats but less so for anxiety-like behavior during alcohol withdrawal; collectively, these data may be helpful in informing experiments designed to investigate chronic alcohol effects on behavioral outcomes.

## 1. Introduction

Alcohol use disorder (AUD), characterized by excessive and compulsive alcohol consumption despite adverse consequences, remains a widespread public health concern. According to the 2023 National Survey on Drug Use and Health (NSDUH), 224.3 million people aged 12 and older reported drinking alcohol at some point in their lifetime, and 16.3 million adults ages 18 and older reported heavy alcohol use (SAMHSA, 2023). Continued excessive alcohol consumption can lead to the development of dependence that is associated with a withdrawal syndrome when alcohol consumption is ceased or substantially reduced. This syndrome includes physical and psychological symptoms that contribute to distress and psychological discomfort (Bokström et al., 1989, 1991; Roelofs, 1985; Roelofs and Dikkenberg, 1987). These negative emotional components of withdrawal, anxiety in particular, are related to relapse during abstinence (Annis et al., 1998; De Soto et al., 1989; Hershon, 1977; Miller and Harris, 2000; Willinger et al., 2002). Bidirectional associations between AUD and chronic pain syndromes also have been reported (Apkarian et al., 2005, 2011; Brennan and Moos, 2005; Egli and Edwards, 2012; Zale et al., 2015). The prevalence of AUD is increased in adult patients suffering from chronic pain conditions, partly due to alcohol’s analgesic properties (Hoffmann et al., 1995), which may be heightened among individuals with alcohol dependence (Brennan and Moos, 2005; Cutter et al., 1976; Kim et al., 2013). The widespread prevalence of AUD and its profound impact on physical and psychological well-being during withdrawal and abstinence underscore the critical need for comprehensive research to unravel the neurobiological mechanisms at play. This highlights the essential role of animal studies in deepening our understanding of these complex phenomena.

Rat models of alcohol dependence often incorporate methods of forced alcohol administration to assess alcohol effects on behavior, while allowing for experimental control of exposure parameters (Avegno and Gilpin, 2019; Gilpin et al., 2008). Chronic alcohol vapor exposure is often used to generate heightened nociception (Avegno et al., 2018; Bertagna et al., 2024; Brandner et al., 2023; Edwards et al., 2012; Secci et al., 2024; Yang et al., 2024) and anxiety-like behavior (Cruise et al., 2025; Ewin et al., 2019; Gilpin et al., 2008; Healey et al., 2023; Kliethermes, 2005; Valdez et al., 2002; Zhao et al., 2007) during withdrawal, but these behavioral phenotypes are not always observed (Bloch et al., 2022; Ehlers et al., 2011). Variables including sex, duration of alcohol exposure, timing of testing following alcohol cessation, and blood alcohol concentration (BACs) may influence the emergence, magnitude, and trajectory of these behaviors in rodents. While nociceptive assays can be repeated over time in the same animal (Edwards et al., 2012), anxiety-like behavioral assays (e.g., elevated plus maze) are not repeatable without generating habituation effects or adding any other confounding factors (e.g., reduced open arms exploration in the EPM) (Cnops et al., 2022; Leussis and Bolivar, 2006). Given this limitation, it would be advantageous from an experimental design standpoint to identify alcohol exposure parameters associated with a higher likelihood of observing anxiety-like behavior during alcohol withdrawal in alcohol-dependent rats, as this would minimize the potential of failed experiments due to lack of a withdrawal-associated behavioral phenotype.

Here, we performed retrospective analysis of data related to anxiety-like and nociceptive behaviors in adult male and female Wistar rats exposed to chronic intermittent alcohol vapor. Our goal was to determine the association between alcohol vapor exposure parameters and behavioral outcomes during acute withdrawal. Our results indicate that vapor exposure parameters (including average BACs over time and length of vapor exposure) are significantly positively correlated with thermal nociception, but not anxiety-like behavior, in CIE rats tested during withdrawal. The results of this analysis add to our understanding of alcohol withdrawal-associated behaviors in rats and may be helpful when planning studies involving alcohol dependence.

## 2. Methods and materials

### 2.1. Animals

All procedures were approved by the Institutional Animal Care and Use Committee of the Louisiana State University Health Sciences Center and were carried out in accordance with the National Institutes of Health guidelines. Adult male and female Wistar rats were received at postnatal day (PND) 56 and double-housed in a humidity- and temperature-controlled (22 °C) vivarium on a 12-h light/dark cycle (lights off at 7 a.m.), with *ad libitum* access to food and water. The experiments in this study were carried out over an extended period, during which animals were sourced from Charles River Laboratories in Raleigh, NC, USA and Kingston, NY, USA. Housing conditions also changed during this period following a laboratory relocation in mid-2023, coinciding with the timing of the Charles River location change. Additional analyses were conducted to determine whether baseline behavioral data differed based on factors including time (reflective of change in rat source location and laboratory relocation), season during which experiments were performed, or individual performing behavioral scoring; no significant differences based on these factors were observed (see Supplementary Fig. 1). All animals were acclimated to housing conditions and light/dark cycles for 1 week before the start of experiments. A total of 258 rats (males: n = 211; females: n = 47) were used across experiments.

### 2.2. Chronic intermittent exposure (CIE) to alcohol vapor

A CIE to alcohol vapor protocol was used to induce dependence in rats. Rats were pair-housed in sealed chambers (La Jolla Alcohol Research, La Jolla, CA, USA) and exposed to alcohol vapor for 14 h per day as previously reported (Avegno and Gilpin, 2019; Gilpin et al., 2008). Blood alcohol levels (BACs) were measured 1–2 times weekly and analyzed using an Analox AM1 analyzer (Analox Instruments, Lunenberg, MA, USA). A range of 150–250 mg/dL was targeted for all rats for a minimum of 4 weeks (across all experiments: 158.5 ± 6.1 mg/dL; mean ± SEM), although individual variability occasionally led to low (<150 mg/dL) or high (>300 mg/dL) BACs. Rats with a BAC >300 mg/dL were removed from vapor overnight, to allow sufficient time for BACs to return to 0 before subsequent alcohol exposure. All behavioral tests occurred 6–8 h following vapor cessation, at a time point when BACs were approximately 0 mg/dL. In some experiments, the length of CIE exposure was extended until the emergence of stable withdrawal hyperalgesia was observed.

### 2.3. Elevated plus maze for anxiety-like behavior

The elevated plus maze test (EPM) was used to test locomotor and anxiety-like behavior during acute alcohol withdrawal. The EPM was a black Plexiglas apparatus consisting of two closed arms (50 cm × 10 cm × 40 cm) and two open arms (50 cm × 10 cm) attached to metal legs elevating the maze 50 cm above the ground. All testing was conducted under dim illumination (approximately 10 lux in the open arms). Rats were placed individually in the center of the maze and allowed 5 min of free exploration. Behavior was recorded with a camera positioned above the maze. The EPM was cleaned thoroughly between subjects using Quatricide. Video scoring was done by an observer blind to the conditions; time spent in open arms was calculated as a percentage of the time spent in the open arms divided by the total time in the open and closed arms (omitting center time). One arm entry was defined as all four paws entering the arm.

### 2.4. Open field for anxiety like-behavior

Open field testing was performed as a second measure of locomotor and anxiety-like behavior during alcohol withdrawal. Open field and EPM testing occurred at the same time of day on different days, with a minimum of 24 h between testing. The open field apparatus consists of a 72 × 72 cm acrylic arena, partitioned into 25 squares, each with a perimeter of 14.2 cm. Enclosed by walls to prevent escape, the arena features a light intensity of 15–20 lux at its center, gradually decreasing to less than 5 lux at the periphery and corners. The animals were put in the corner of the open field and allowed 5 min to explore the arena. The amount of time each rat spent exploring the center (defined by exploring boxes that do not border the walls of the apparatus) and periphery (defined by exploring boxes that border the walls of the apparatus) was quantified by an observer blind to the treatment. The time spent in the center was calculated in seconds, and lower scores in this measure were interpreted as higher anxiety-like behavior. The number of lines crossed was calculated and interpreted to reflect non-specific locomotor activity.

### 2.5. Hargreaves test for thermal nociception

On test days, rats were allowed 30 min to acclimate to the testing environment and then placed in a 4×8×5 inch clear Plexiglass enclosure on top of a glass panel suspended 8 inches above the tabletop. After a brief habituation period, each hindpaw was stimulated by a halogen light heat source from an IITC model 309 Hargreaves apparatus (75 % of maximum light intensity; IITC Life Sciences, Woodland Hills, CA, USA). Latency to withdraw the hindpaw was measured twice for each hindpaw in alternating order, with at least 1 min between measurements. A 20-s cutoff was used to prevent tissue damage in unresponsive subjects. The average of latency measurements across the 4 trials provided the thermal nociception score for each rat.

### 2.6. Data and statistics

Data were analyzed with one-way ANOVA, two-way ANOVA and simple linear regression. For all analyses, significance was assigned at *p* < 0.05. Data are presented as mean ± SEM. All statistical analyses were completed with GraphPad Prism 10.4.1 (GraphPad Software, San Diego, CA USA).

## 3. Results

### 3.1. Anxiety-like behavior during acute alcohol withdrawal using the elevated plus maze

**Fig. 1.**
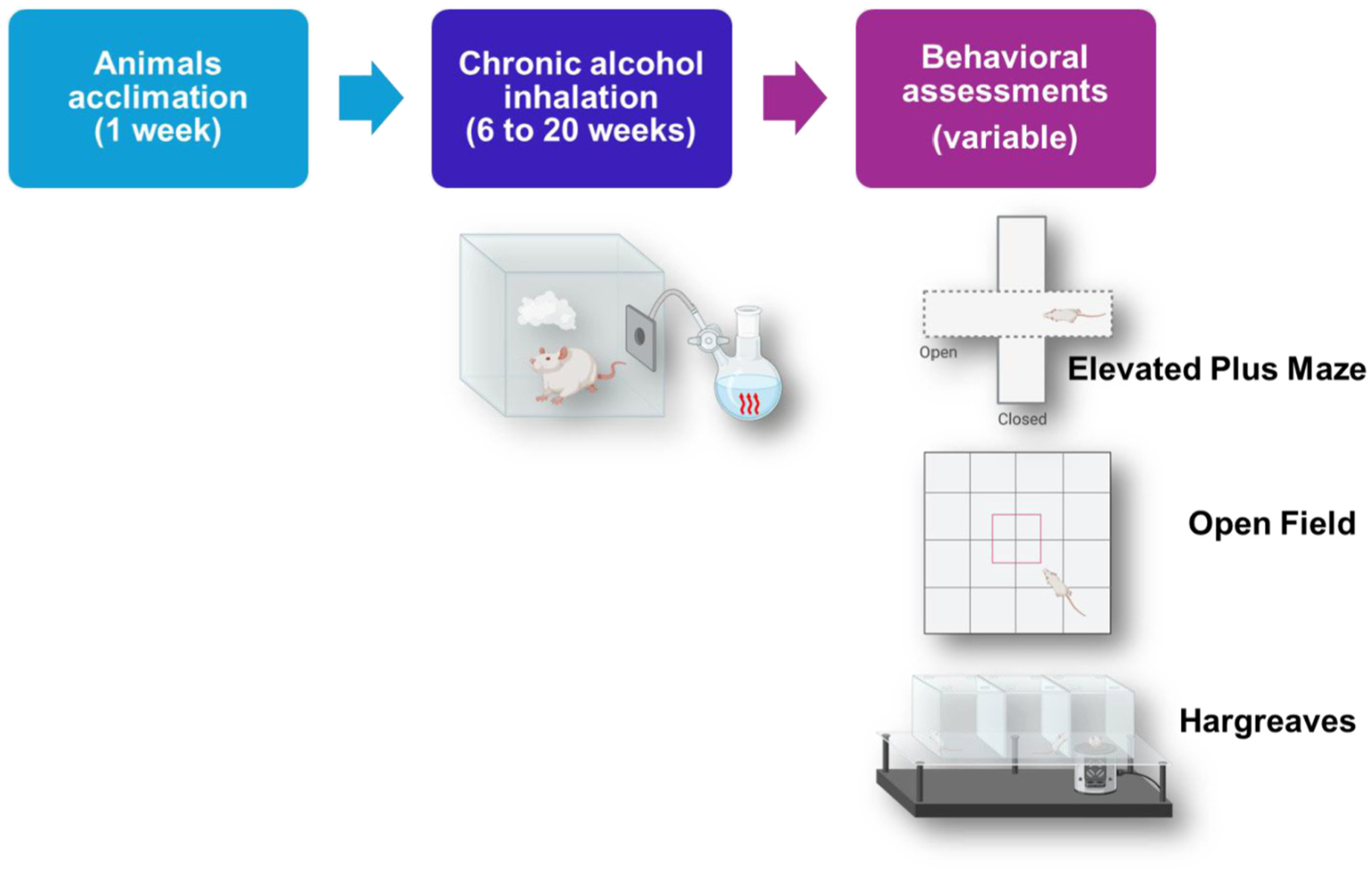
Experimental Timeline. Adult male and female Wistar rats were allowed one week of acclimation in our facilities, then underwent chronic intermittent alcohol inhalation (14 h each day) for a minimum of 6 weeks to a maximum of 20. During acute withdrawal, the animals were then tested for pain-like behaviors, such as Hargreaves for thermal nociception and Elevated Plus Maze and Open Field for anxiety-like behavior assessments.

Male and female Wistar rats were exposed to chronic alcohol inhalation for durations ranging from 6 to 20 weeks, as shown in Fig. 1. Multiple cohorts were included in a retrospective analysis of vapor exposure parameters and withdrawal-associated behaviors. Variables included in our analysis were the total length of vapor exposure (defined as the number of weeks of CIE, beginning when a rat’s BAC was first >150 mg/dL), the average BAC across vapor exposure, and whether rats ever achieved a BAC >300 mg/dL.

**Fig. 2.**
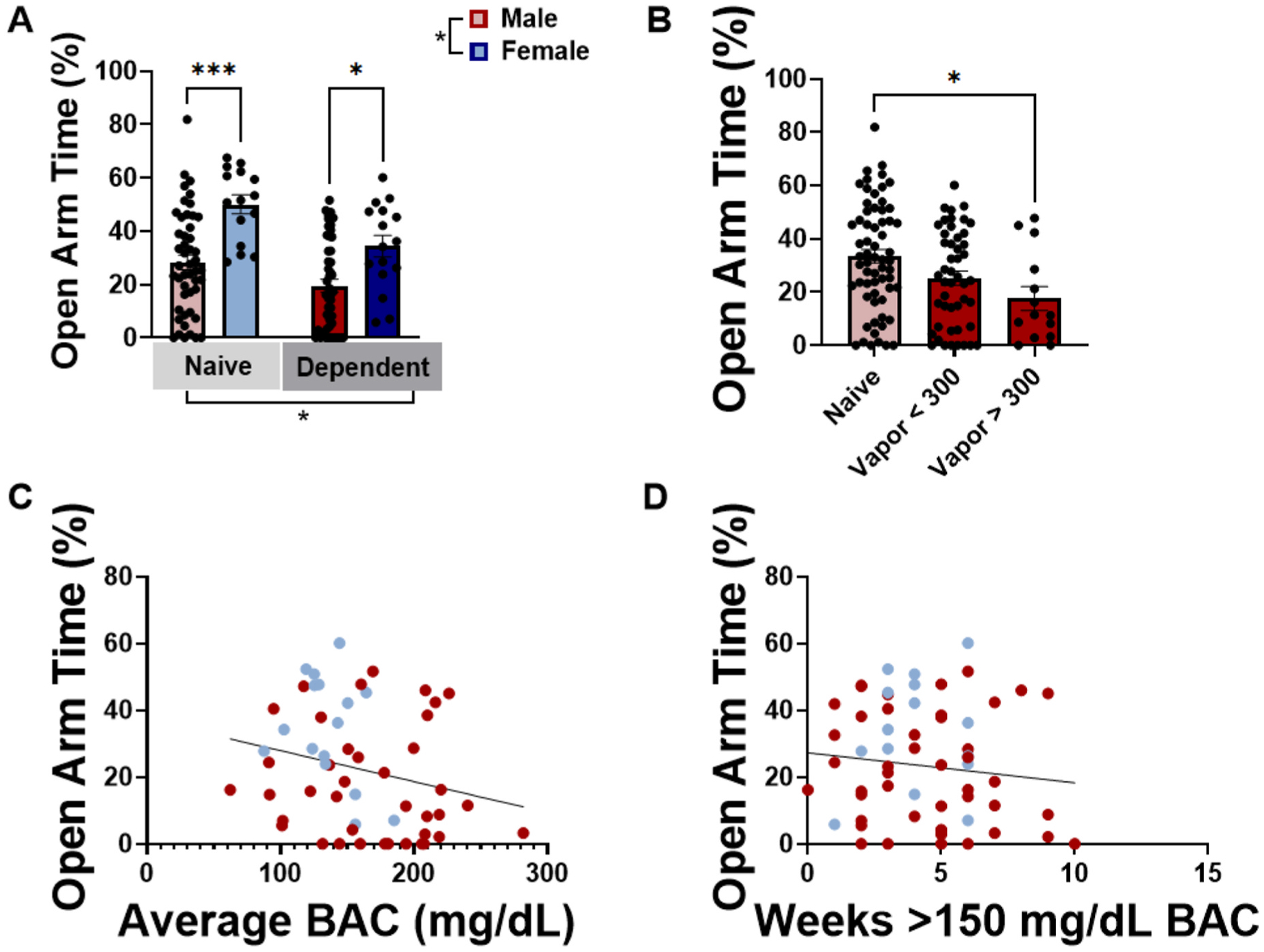
Vapor Exposure Parameters and Elevated Plus Maze Data. (**A**) Time spent in the open arm of an elevated plus maze (expressed as a percentage of total open and closed arm time) in alcohol-naïve and dependent male (red) and female (blue) Wistar rats following acute withdrawal from chronic alcohol vapor exposure. (**B**) Open arm time in rats exposed to no alcohol (“Naive”), compared to CIE rats with no history of BACs >300 mg/dL (“Vapor <300”) and rats that achieved a BAC >300 mg/dL at least once (“Vapor >300”). (**C**) Correlation between average BACs and percentage time spent in the open arm. Data from male rats shown in red; females in blue. (**D**) Correlation between the number of weeks during which a rat had a BAC >150 mg/dL and percentage time spent in the open arm of an elevated plus maze. Data in (**A**) and (**B**) shown as mean ± SEM; *p < 0.05, ***p < 0.001.

We first compared anxiety-like behavior, as indicated by time spent in the open arm of an elevated plus maze, in male and female rats. A two-way ANOVA revealed a significant main effect of CIE (F_(1,123)_ = 11.4, *p* = 0.0010) and a significant main effect of sex (F_(1,123)_ = 25.8, *p* < 0.0001) on open arm time. Tukey’s multiple comparisons post hoc test indicated significant differences between male naïve vs female naïve rats (*p* = 0.0003), female naïve vs male vapor rats (p < 0.0001), and male vapor vs female vapor rats (*p* = 0.0199; Fig. 2A). A similar relationship was observed when comparing time spent in the closed arm of the EPM (significant main effect of CIE (F_(1,123)_ = 9.94, *p* = 0.002) and a significant main effect of sex (F_(1,123)_ = 21.0, *p* < 0.0001), two-way ANOVA, Supplementary Fig. 2A), number of closed arm entries (significant main effect of CIE (F_(1,123)_ = 6.67, *p* = 0.011), two-way ANOVA, Supplementary Fig. 2B), and number of open arm entries (significant main effect of CIE (F_(1,123)_ = 18.7, *p* < 0.0001) and a significant main effect of sex (F_(1,123)_ = 19.9, *p* < 0.0001), two-way ANOVA, Supplementary Fig. 2C).

We next separated CIE rats into groups based on whether a high BAC (≥300 mg/dL) was ever achieved during the vapor exposure and compared open arm time between CIE rats and alcohol-naive controls. One-way ANOVA revealed a significant main effect of CIE (F_(2,122)_ = 5.1, *p* = 0.0075), and Tukey’s multiple comparisons post hoc test revealed a significant difference between alcohol-naïve (Air) controls and CIE rats with a BAC >300 mg/dL (*p* = 0.0157; Fig. 2B). Rats with a history of higher BACs (>300 mg/dL) tended to show lower open arm time than rats with lower BACs (<300 mg/dL), although this did not reach statistical significance.

Linear regression analysis between average BACs across the duration of vapor exposure and time spent in open arms showed no significant relationship between the two parameters (simple linear regression, slope = −0,093, R^2^ = 0.053, *p* = 0.087; Fig. 2C). Finally, no correlation was found between open arm activity and the total number of weeks the BACs exceeded 150 mg/dL (simple linear regression, slope = 0.90, R^2^ = 0.014, *p* = 0.35; Fig. 2D). Collectively, these analyses indicate that several vapor parameters, including average BACs and time spent >150 mg/dL, are not significantly associated with behavioral outcomes in the elevated plus maze.

### 3.2. Anxiety-like behavior during acute alcohol withdrawal using the open field test

**Fig. 3.**
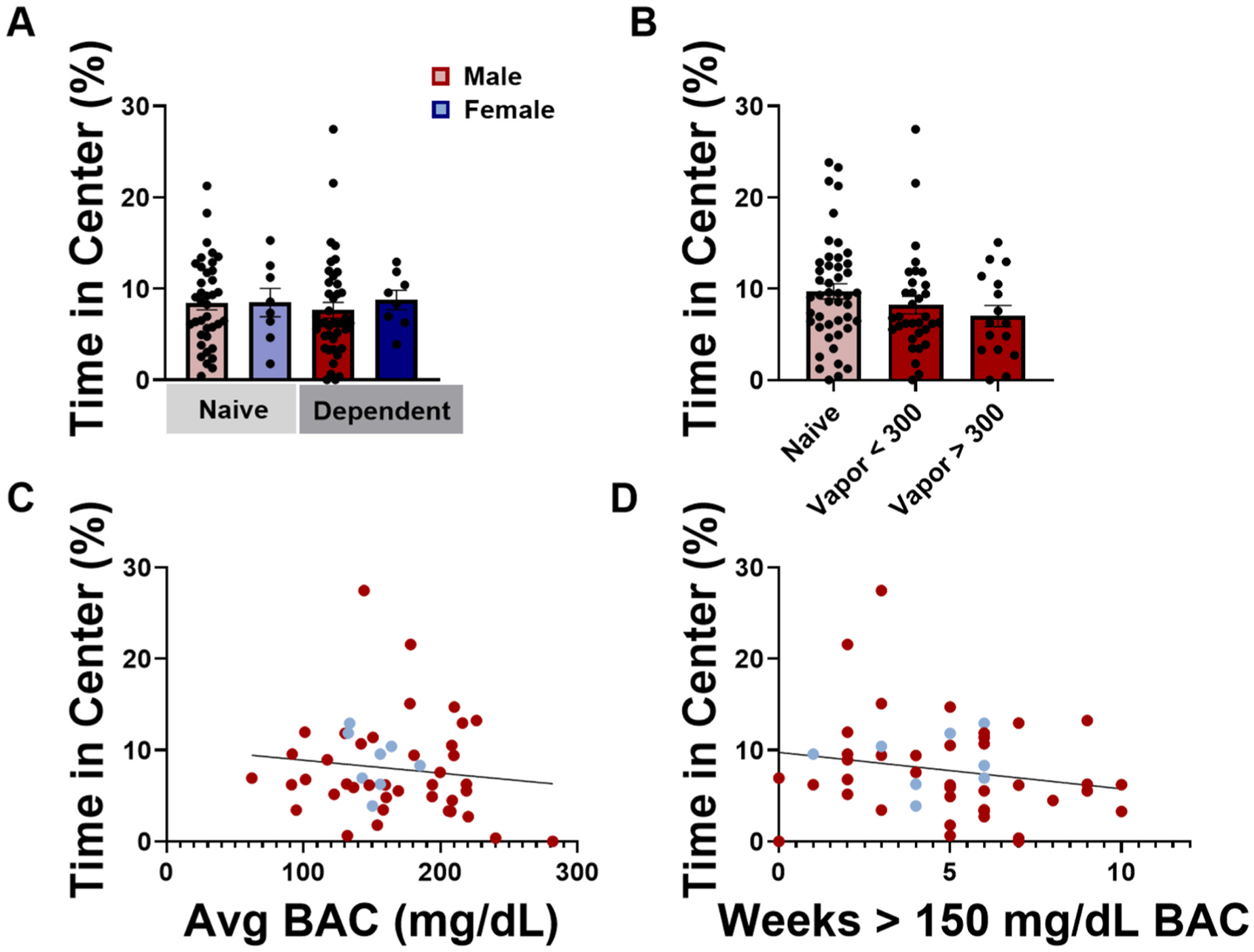
Vapor Exposure Parameters and Open Field Data. (**A**) Time spent in the center of an open field (expressed as a percentage of total center and periphery time) in alcohol-naïve and dependent male (red) and female (blue) Wistar rats tested during acute withdrawal. (**B**) Time spent in the center of the open field in rats exposed to no alcohol (“Naïve”), compared to CIE rats with no history of BACs >300 mg/dL (“Vapor <300”) and rats that had achieved a BAC >300 mg/dL at least once (“Vapor >300”). (**C**) Correlation between average BACs and percentage time spent in the center of the open field arena. Data from male rats shown in red; females in blue. (**D**) Correlation between total number of weeks during which a rat had a BAC >150 mg/dL and percentage time spent in the center of the open field arena. Data in (**A**) and (**B**) shown as mean ± SEM.

Mirroring the earlier results, rats were evaluated in the open field to assess spontaneous exploration during acute withdrawal from alcohol (defined as the time when BACs would be 0 mg/dL, approximately 6–8 h after the last vapor exposure). Unlike the EPM results, we found no sex differences for time spent in the center of the arena, nor significant differences between alcohol-naive and dependent rats (Fig. 3A). We also found no significant differences in time spent in the arena center whenever BACs were either higher or lower than 300 mg/dL (*p* > 0.05; Fig. 3B).

Correlation analysis indicated no relationship between time spent in center of arena and average BACs (Fig. 3C), nor between time spent in center of arena and the number of weeks in which BACs were above 150 mg/dL (Fig. 3D). These results suggest that, despite there being an expectation of anxiety-like behavior (i.e., lower center time) manifesting during acute alcohol withdrawal, this was not consistently observed in CIE rats in an open field test, and the measured vapor exposure parameters do not have a significant correlation with behavioral outcomes.

### 3.3. Thermal nociception during acute alcohol withdrawal in male and female Wistar rats

**Fig. 4.**
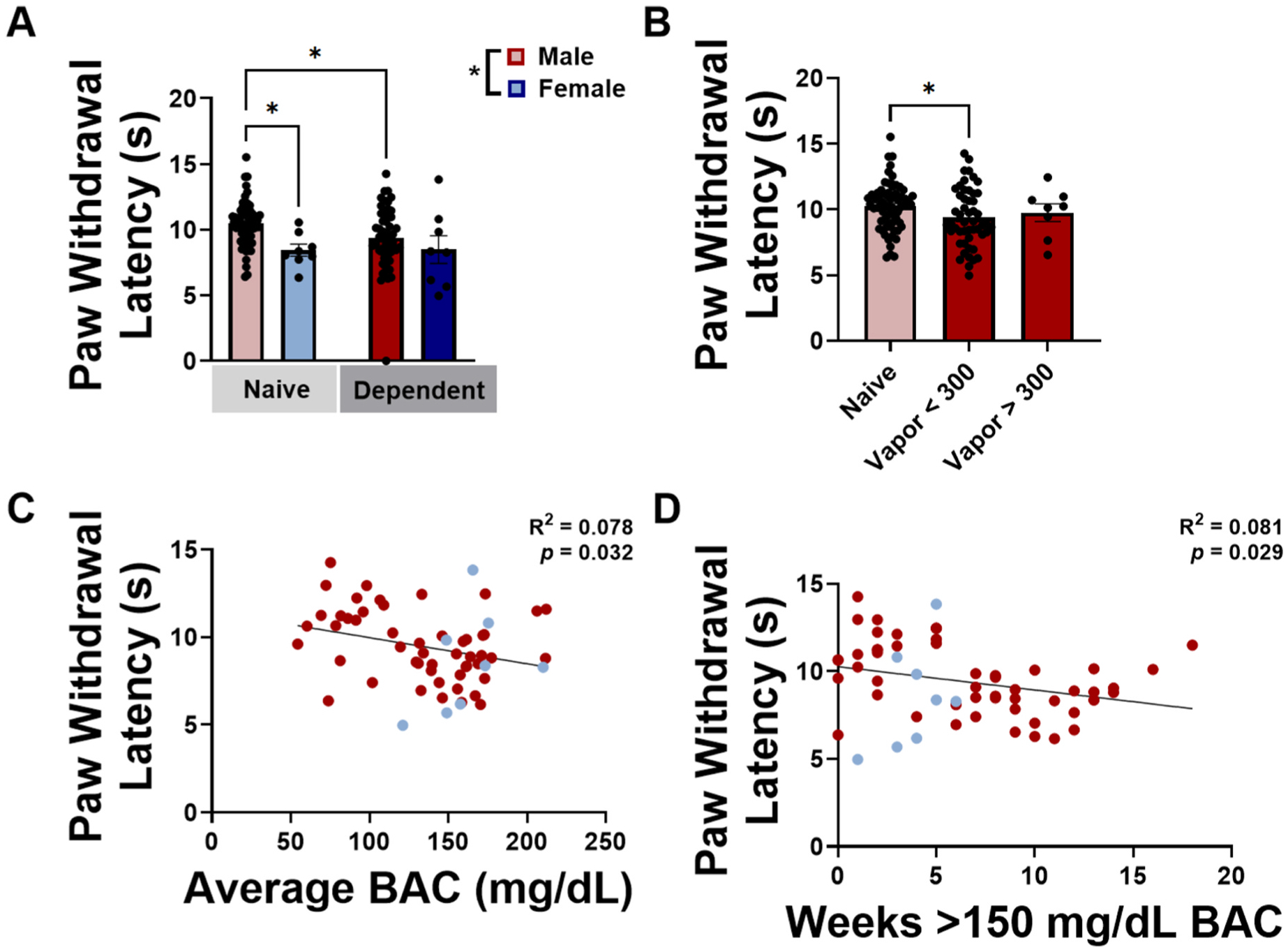
Correlations Between Vapor Exposure Parameters and Thermal Nociception Data. (**A**) Hindpaw withdrawal latency during Hargreaves testing in alcohol-naïve and dependent male (red) and female (blue) Wistar rats tested during acute withdrawal. (**B**) Hindpaw withdrawal in rats exposed to no alcohol (“Naïve”), compared to CIE rats with no history of BACs >300 mg/dL (“Vapor <300”) and rats that had achieved a BAC >300 mg/dL at least once (“Vapor >300”). (**C**) Correlation between average BACs and hindpaw withdrawal latency. (**D**) Correlation between the number of weeks during which a rat had a BAC >150 mg/dL and hindpaw withdrawal latency. Data in (**A**) and (**B**) shown as mean ± SEM; *p < 0.05.

We used the Hargreaves test to measure thermal hypersensitivity in male and female Wistar rats during acute alcohol withdrawal. A two-way ANOVA revealed a significant main effect of sex on the hindpaw withdrawal latency (F_(1,127)_ = 7.15, *p* = 0.0085; Fig. 4A). Because we observed a main effect of sex, we performed separate analyses in males and females to determine the effects of alcohol within each sex. A comparison of hindpaw withdrawal latency revealed a significant difference between naïve and alcohol-dependent males (*t*_95.8_ = 2.85 *p* = 0.0054, Welch’s *t*-test; Fig. 4A), though no significant difference was found when comparing females (*t*_9.5_ = 0.042, *p* = 0.97). This indicates that acute alcohol withdrawal causes increased hyperalgesia in adult male rats, similarly to what our lab previously described in adolescents (Secci et al., 2024) and adults (Avegno et al., 2018).

When comparing hindpaw withdrawal latency values between rats separated by a history of achieving a BAC >300 mg/dL, we found that elevated BACs do not necessarily predict a thermal hyperalgesia phenotype. One-way ANOVA indicated no significant effect of CIE (F_(2,125)_ = 2.87, *p* = 0.060), but Tukey’s multiple comparisons post hoc test revealed a significant difference between alcohol-naïve controls and CIE rats without a history of achieving high BACs (<300 mg/dL; *p* = 0.048; Fig. 4B). This suggests that thermal hypersensitivity mainly occurs when blood alcohol levels do not reach excessive levels.

A comparison between average BACs across the duration of vapor exposure and hindpaw withdrawal revealed a weak but significant negative relationship, whereby higher average BACs were associated with decreased withdrawal latency (simple linear regression, slope = −0.015, R^2^ = 0.078, *p* = 0.032; Fig. 4C). Similarly, a comparison of paw withdrawal latency and the total number of weeks during which a given rat’s BAC was >150 mg/dL revealed a significant correlation (simple linear regression, slope = 0.060, R^2^ = 0.081, *p* = 0.029; Fig. 4D). These results suggest that vapor parameters, including higher alcohol concentrations and longer exposure durations, are associated with the occurrence of pain-like behaviors.

## 4. Discussion

Repeated cycles of alcohol intoxication and abstinence produce allostatic changes, including downregulated mesolimbic reward function coupled with recruitment and sensitization of extended amygdala stress systems (e.g., CRF, dynorphin, noradrenergic signaling), which together blunt reward responsiveness and elevate stress/affective pain (Koob et al., 2001; Koob and Vendruscolo, 2025; Merlo Pich et al., 1995; Walker et al., 2012; Wise and Koob, 2014). The resulting heightened emotional and physical pain during periods of absence, a state referred to as alcohol withdrawal-induced hyperkatifeia (Koob and Vendruscolo, 2025), is hypothesized to drive alcohol addiction (Koob, 2021). The neurobiological mechanisms linking alcohol withdrawal to pain and anxiety remain poorly characterized, and animal models are essential tools for investigating these processes. While some voluntary drinking studies in rats report high levels of alcohol intake (summarized in (Carnicella et al., 2014)) and have observed behavioral phenotypes including aversion-resistant drinking (Hopf et al., 2010; Seif et al., 2013) and deficits in working memory (George et al., 2012), studies often utilize involuntary alcohol exposure to study alcohol withdrawal-induced behavioral phenotypes and neurobiological correlates, as these allow for more precise experimental control over exposure patterns.

Human studies indicate associations between binge drinking and the onset of anxiety, while people living with co-occurring mental disorders and AUD are at elevated risk for adverse outcomes, cancers, and mortality (Carr et al., 2024; Zhao et al., 2023). A recent comprehensive parametric alcohol study revealed that even at an average consumption of 20 g/day (equivalent to approximately one large beer), the risk of developing an AUD is nearly threefold that of current non-drinkers, and the risk of AUD-associated mortality is approximately double that of current non-drinkers (Carr et al., 2024). In animal models, repeated binge-like exposures (those that produce high peak BACs) can be more damaging to some endpoints (e.g., liver injury, neuroinflammation) than continuous low-level exposure with the same cumulative alcohol mass, highlighting the impact of pattern of administration and the peak BAC achieved on physiological outcomes (Kuzmin et al., 2012). Considering the existing data in humans and rodents correlating alcohol exposure patterns on biological outcomes, our goal was to assess whether similar relationships relevant to withdrawal-associated behavioral outcomes are observed in rats.

Here, we report that chronic alcohol exposure leads to higher anxiety-like behavior during withdrawal in male and female Wistar rats, as evidenced by reductions in time spent in the open arms of an elevated plus maze, although this was not consistently replicated in the open field test, another assay often used for testing anxiety-like behavior in rodents (File et al., 2004; Snyder et al., 2021). While the EPM and open field test are two common measures of anxiety-like behavior, both are considered to capture different constructs, with EPM more sensitive to approach-avoidance conflict, and open field capturing general locomotion and thigmotaxis (Carola et al., 2002; Chen et al., 2024; Ramos et al., 2008). Performance in one behavioral assay is not consistently predictive of performance in the other, as shown in our analysis and in other published studies (Sudakov et al., 2013; Turri et al., 2001). While we observed a significant main effect of CIE on time spent in the open arm of an EPM (Fig. 2B), posthoc analysis revealed a significant difference between rats with a history of very high BACs (>300 mg/dL), and not CIE rats without a history of 300 mg/dL. The aim of the present study was not to validate either behavioral model, but rather to examine how variation in exposure parameters relates to the expression of withdrawal-associated affective behaviors within a chronic intermittent ethanol vapor paradigm. In this context, anxiety-like behaviors were more evident in animals that achieved higher BACs, consistent with prior reports indicating that affective manifestations of ethanol withdrawal may depend on attaining a sufficient level of dependence. Accordingly, the lack of detectable anxiety-like behavior in lower-BAC dependent rats may reflect variability in withdrawal expression rather than an inherent limitation of the experimental approach.

While we find a significant effect of sex and excessive blood alcohol levels on behavior in the elevated plus maze, there were no significant correlations between the vapor exposure parameters we examined and anxiety-like behavior. Although alcohol withdrawal is often associated with increased anxiety like behavior in rats, effects in the open field are not consistently observed. The absence of an open field anxiety phenotype in our dependent rats is consistent with previous reports and likely reflects behavioral and test specific factors (Ewin et al., 2019). Additional experimental parameters, such as withdrawal timepoint or route of alcohol administration, may be more influential on behavioral outcomes. Previous studies suggest that timing of behavioral assays during withdrawal influences the onset of anxiety-like behaviors. Our tests were conducted in acute withdrawal (6–8 h after alcohol vapor cessation), a timepoint at which other studies have reported increased anxiety-like behavior (Baldwin et al., 1991; File et al., 1991; Kampov-Polevoy et al., 2000). Other studies demonstrated a withdrawal phenotype during protracted withdrawal (e.g., at 24 hr (Ewin et al., 2019; Pandey et al., 1999) or 7 days after cessation (Lal et al., 1991)). These variations likely stem from differences in alcohol exposure duration (Valdez et al., 2002, 2003). The expression of anxiety-like behaviors, both at baseline and during alcohol withdrawal, is also influenced by rat strains. Basal differences in anxiety-like behavior in an EPM have been reported, with Wistar Kyoto rats demonstrating less exploratory behavior than Wistar or Fisher F344 rats (Shepard and Myers, 2008). Among Wistar rats, variability in EPM exploratory behavior rats has been reported (Liebsch et al., 1998); similarly, we report variability in open arm time of alcohol-naïve Wistar rats (Fig. 2A). In alcohol-exposed adult male Long-Evans rats, repeated cycles of CIE vapor produce significant anxiety-like phenotypes, as evidenced by decreased open-arm exploration in the elevated plus maze and increased avoidance behaviors in the successive alleys test during acute withdrawal (Ewin et al., 2019; Valdez et al., 2002). In the same strain, early withdrawal from CIE exposure increases anxiety-like behaviors (i.e., EPM) regardless of sex (Morales et al., 2015). P rats also show strain-dependent withdrawal anxiety in these tests, highlighting the role of genetics in shaping affective responses (Kampov-Polevoy et al., 2000). Taken together, EPM- and OFT-based measures capture withdrawal-induced anxiety across strains, while emphasizing meaningful differences in intensity, timing, and behavioral expression that must be considered when designing experiments.

Our analysis pointed to a relationship between alcohol vapor exposure parameters and nociceptive behavior during alcohol withdrawal. Meta-analyses in humans report positive correlations between alcohol dose and analgesia, as well as possible correlations between analgesic effect and alcohol dependence in individuals with persistent pain (Jochum et al., 2010; Thompson et al., 2017). We previously reported a relationship between BACs and paw withdrawal thresholds of adolescent male and female alcohol-exposed rats (Secci et al., 2024). Here, we report increases in thermal hindpaw sensitivity (i.e., hyperalgesia) in alcohol-dependent male and female rats during withdrawal, as well as significant correlations between paw withdrawal thresholds and average BACs and total time in vapor. We note, however, that the correlation observed was relatively weak, with R^2^ values below 0.1, indicating that vapor exposure parameters are not the sole predictors of variance in thermal nociceptive behavior during alcohol withdrawal. Analysis of hindpaw withdrawal latency among groups separated by highest BAC achieved (i.e., whether rats ever had a BAC >300 mg/dL) revealed lower mean withdrawal thresholds in rats with a lower BAC history (<300 mg/dL) compared to those with higher peak intoxication levels (>300 mg/dL; Fig. 4B). We posit that reflex-based measures such as hindpaw withdrawal do not solely depend on sensory detection of noxious stimuli but also on central processing and motor execution. During acute withdrawal (e.g., from very high BACs), competing effects of CNS hyperexcitability (which drives hyperalgesia) and motor impairment (which can alter the animal’s ability to perform a withdrawal response) might reduce or blunt the apparent nociceptive behavior. This pattern of neuroadaptations could explain why there is no simple positive correlation between BAC and hindpaw withdrawal thresholds in this case (Dong et al., 2025). Moreover, moderate increases in BACs are associated with pain threshold increases, but effects on pain intensity and thresholds depend on dose and context, suggesting a complex dose–response curve rather than monotonic increases at all levels (Thompson et al., 2017).

Chronic alcohol vapor exposure leads to increases in pain-like behaviors that emerge during withdrawal, often within hours to days after cessation, allowing for standardized testing windows. Emergence of hyperalgesia has been shown to persist for weeks after vapor cessation, particularly when alcohol exposure occurs during adolescence. Mechanical (Bertagna et al., 2024; Obray et al., 2025) and thermal (Secci et al., 2024) hyperalgesia has been observed up to 6 weeks following the end of vapor exposure during adolescence in mice and rats. Fewer studies have assessed nociception into protracted withdrawal following vapor exposure in adulthood, though one study in mice reported recovery of mechanical hyperalgesia following 3 weeks (males) or 4 weeks (females)(Bertagna et al., 2024). Differences in the timing of onset and duration of hyperalgesia may be related to alcohol exposure parameters or other variables across labs and studies. One recent study reported a correlation between mechanical and thermal nociception and blood alcohol concentrations in mice (particularly females, rather than males), though BACs on average were higher in females compared to males (Brandner et al., 2023). Inter-individual variability in alcohol responses, combined with technical inconsistencies in vapor exposure systems, can affect BACs; therefore, standardizing these parameters is critical for consistent behavioral assessment. Preclinical studies involving other rat strains demonstrated alcohol-associated changes in pain-like behaviors, though direct vapor exposure models in these strains are less common. In adult male Long-Evans rats, repeated cycles of CIE over multiple weeks produced robust mechanical and thermal hyperalgesia upon withdrawal, as evidenced by significant reductions in paw withdrawal thresholds and latencies relative to air-exposed controls (Fu et al., 2021). Moreover, in Sprague-Dawley rats, one of the strains most commonly used in preclinical alcohol research, excessive consume of alcohol through self-administration induced physical dependence and mechanical and thermal hypersensitivity (Fu et al., 2015).

The retrospective analysis reported here indicates a relationship between vapor exposure parameters and thermal nociception in male and female rats, suggesting that vapor parameters may be useful in predicting the onset of hyperalgesia in experimental rats. This was not the case for anxiety-like behavior, which was not significantly correlated with vapor exposure parameters. These data may serve as a useful resource for researchers planning experiments incorporating chronic alcohol vapor exposure in rats.

## Supplementary Material

1

## Appendix A. Supplementary data

Supplementary data to this article can be found online at https://doi.org/10.1016/j.alcohol.2026.01.157.
