# Supplementary material for: Chronic alcohol exposure parametric effects on anxiety- and pain-related behaviors in adult rats
Source: Alcohol. Author manuscript; Available in PMC 2026 Jul 10. (PMC13353107; doi:10.1016/j.alcohol.2026.01.157)
Supplement: 1 [file NIHMS2190231-supplement-1.docx]

**Supplementary Figure 1: Comparison of EPM Scores Based on Experimental Factors.** (**A**) Time spent in the open arm of an elevated plus maze (expressed as a percentage of total open and closed arm time) in all alcohol-naïve rats included in analysis in Figure 2. Data separated based on the season during which experiments occurred. No difference between groups was found (F_(3, 65)_ = 0.19, *p* = 0.90; one-way ANOVA). (**B**) Time spent in the open arm of an elevated plus maze (expressed as a percentage of total open and closed arm time) in all alcohol-naïve rats included in analysis in Figure 2. Data separated based on two separate experimenters performing EPM scoring; groups also correspond to other experimental factors (reflective of change in rat source location and laboratory relocation). No difference between groups was found (t_(69.7)_ = 0.084, *p* = 0.93; two-tailed *t*-test). Data in (**A**) and (**B**) shown as mean ± SEM.


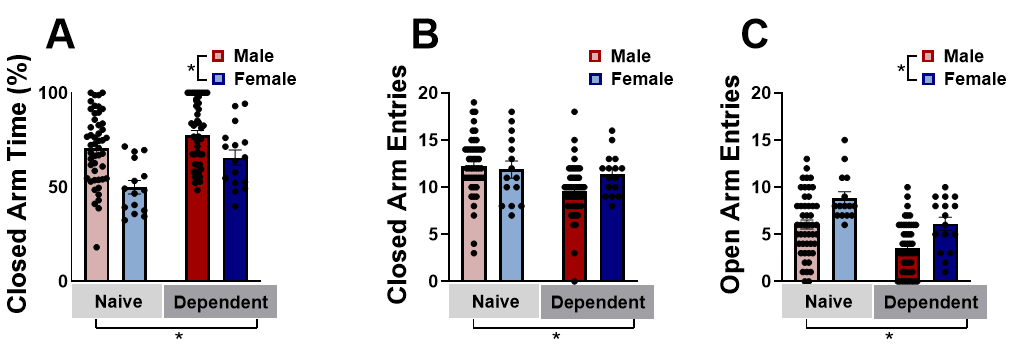
**Supplementary Figure 2: Additional EPM Parameters across Cohorts.** (**A**) Time spent in the closed arm of an elevated plus maze (expressed as a percentage of total open and closed arm time) in all alcohol-naïve and dependent rats included in analysis in Figure 2. Total number of closed (**B**) and open (**C**) arm entries in all alcohol-naïve and dependent rats included in analysis in Figure 2. Data from males shown in red; females in blue. Data shown as mean ± SEM. **p* < 0.05, two-way ANOVA.
